# Assessment of Culture-Negative Neonatal Early-Onset Sepsis: Risk Factors and Utility of Currently Used Serum Biomarkers

**DOI:** 10.3390/children12030355

**Published:** 2025-03-13

**Authors:** Diana Iulia Vasilescu, Adriana Mihaela Dan, Laura Andreea Stefan, Sorin Liviu Vasilescu, Vlad Dima, Monica Mihaela Cîrstoiu

**Affiliations:** 1 Doctoral School, “Carol Davila” University of Medicine and Pharmacy, 020021 Bucharest, Romania; 2 Department of Neonatology, Emergency University Hospital Bucharest, 050098 Bucharest, Romania; 3 Faculty of Medicine, “Carol Davila” University of Medicine and Pharmacy, 020956 Bucharest, Romania; 4 Department of Neonatology, Marie Curie Children’s Hospital, 077120 Bucharest, Romania; 5 Department of Obstetrics and Gynecology, Emergency University Hospital Bucharest, 050098 Bucharest, Romania; 6 Department of Neonatology, Filantropia Clinical Hospital, 011132 Bucharest, Romania

**Keywords:** culture-negative early neonatal sepsis, risk factors, C-reactive protein, biomarkers, antibiotic stewardship

## Abstract

Introduction: Neonatal sepsis is a severe and life-threatening condition caused by pathogens in the systemic circulation within the first 28 days of life. The classical definition of neonatal sepsis implies positive central cultures, but recent findings discuss culture-negative sepsis (clinical sepsis associated with laboratory findings). Since infected neonates initially express few non-specific clinical signs and there are unreliable biochemical markers to identify sepsis in the early stages, it is essential to improve the accuracy of diagnosis and reduce unnecessary antibiotic exposure. Objective: Our study aims to assess the influence of risk factors and the utility of currently used biomarkers in culture-negative neonatal early-onset sepsis (CN-EOS). Materials and methods: We performed a retrospective study at Bucharest University Hospital, which included 131 preterm and term newborns at risk for EOS admitted in the Neonatal Intensive Care Unit (NICU) over 12 months. The neonates included were classified into two groups: confirmed negative-culture early-onset sepsis (CN-EOS) and suspected early-onset sepsis (S-EOS). Patients from both groups received antibiotic therapy from the first day of life; the type and duration of antibiotic therapy were different in the two groups. For all the patients, we measured C-reactive protein (CRP), procalcitonin (PCT) and white blood count (WBC) at birth and after 72 h, tested blood culture in the first 24 h of life and correlated the results with clinical signs and prenatal risk factors. Categorical variables were presented as frequencies and percentages, while the continuous variables were the mean and the standard deviation. The differences between the continuous variable groups were determined by Student’s *t*-test or the Mann–Whitney U test, whereas for the categorical variables, the Chi-square test (X^2^) was employed. The performance of laboratory biomarkers (CRP and PCT) in diagnosing confirmed EOS was calculated. All the tests were statistically significant at a *p*-value < 0.05. Results: The findings support the significance of low birth weight and gestational age and low Apgar scores as potential indicators for EOS; PROM diagnosed with chorioamnionitis and smoking during the pregnancy were also important predictive risk factors. Respiratory signs, such as apnea and respiratory distress syndrome, were most encountered in the clinical evaluation of infants with CN-EOS. Inflammatory markers were inconsistent in CN-EOS cases, proving that they are not reliable enough for initiating, continuing or stopping antibiotic therapy. Conclusions: Culture-negative neonatal sepsis remains a significant challenge for the neonatologist, since the time elapsed between the moment sepsis is suspected and the initiation of empirical therapy can make the difference between survival and death. Continued efforts are needed to develop more reliable and effective diagnostic tools for timely and appropriate intervention.

## 1. Introduction

Neonatal sepsis is a severe and life-threatening condition, caused by the presence in the systemic circulation of various microbial organisms, such as bacteria, viruses or fungi, within the first 28 days of life [1].

The incidence of neonatal sepsis is not evenly spread worldwide, with higher rates observed in developing countries, where access to healthcare and hygiene practices may be suboptimal. High-income countries report an incidence of 1 to 5 cases in 1000 births, while in low-income countries, it can increase up to 20 times [2,3].

Despite advancements in the medical field, neonatal sepsis is still a critical health issue affecting newborns globally, representing the leading cause of morbidity and mortality [4]. Every year, more than one million newborns worldwide die due to this infection [5].

The classical definition of neonatal sepsis implies positive central cultures, usually samples from the bloodstream or cerebrospinal fluid [6]. Since the low sensitivity of blood cultures has been proven, especially in newborns (a rate of only 35–50% positive results), most experts emphasize the importance of not excluding neonatal sepsis in the absence of pathogen identification [7]. Moreover, recent findings discuss culture-proven and culture-negative sepsis (clinical sepsis based on laboratory findings and clinical status) [8].

Diagnosis of neonatal sepsis remains a challenge for the neonatologist since there is a large overlap between the clinical pattern in early-onset sepsis and difficult perinatal transition [9].

In clinical practice, newborns are also treated with antibiotics for “probable” sepsis without isolating a pathogen; exact data regarding the number of treated infants for suspicion are not available, as epidemiological studies usually do not include this category of patients, focusing only on culture-proven sepsis. A recent publication reported that the number of infants treated for culture-negative sepsis exceeded the number of newborns receiving antibiotic therapy for culture-proven sepsis [10]. This approach represents a significant increase in antibiotic exposure; according to Lacetta et al., almost 10% of newborns are treated with antibiotics for suspected EOS [11]. Negative-culture sepsis requires broad-spectrum antibiotic therapy to cover most of the pathogens, compared to sepsis with identified etiology, in which an antibiogram allows the use of targeted drugs according to sensitivity. It is important to improve the timing and accuracy of diagnosis to protect patients against polypharmacy. Negative short- and long-term consequences of antibiotic therapy are gut dysbiosis and necrotizing enterocolitis, allergies, increased antibiotic resistance and diabetes [12].

Some potential reasons for the high number of neonates treated for culture-negative sepsis are the use of antibiotic therapy during pregnancy and the perinatal period, inadequate sample for blood culture, low levels of bacteremia and even the possibility of overdiagnosis of sepsis among these newborns [13].

Neonatal sepsis is classified, based on the time of onset, as early-onset sepsis (EOS), occurring within the first 72 h of life, and late-onset sepsis (LOS), occurring after 72 h [14].

Early diagnosis and prompt initiation of appropriate antibiotic therapy are crucial in reducing complications of sepsis and improving outcomes for affected infants [15]. Neonatal sepsis can rapidly progress and lead to severe complications, such as multiorgan dysfunction, shock and death [16].

To improve the accuracy of diagnosis and management in neonatal sepsis, researchers have explored various biomarkers, with a particular focus on the C-reactive protein (CRP). CRP is an acute-phase reactant protein produced by the liver in response to inflammation. Elevated levels of CRP have been found in EOS; however, it is important to note that other factors can influence its values, such as the mode of delivery and postnatal age [17]. Even so, CRP is the most commonly used marker in neonatal sepsis (early or late), although it has limitations and can lead to false positive or false negative results in clinical practice.

Another commonly used biomarker for diagnosis and monitoring neonatal sepsis is procalcitonin (PCT), a precursor of the hormone calcitonin. PCT has demonstrated promising performance in differentiating bacterial infections from non-infectious inflammatory conditions and in monitoring the response to antimicrobial therapy [18].

The use of both CRP and PCT to predict or support the diagnosis of negative-culture sepsis could theoretically increase the accuracy and rapidity of intervention. Unfortunately, different clinical trials comparing the sensitivity and specificity of these two tests separately and altogether did not prove a clear benefit to lead to a strong recommendation on when or how to use them.

Therefore, in the absence of positive cultures, predicting EOS and differentiating it from clinical conditions with similar presentations remains a challenge [19]. 

Novel approaches, such as inflammatory cytokine flow cytometry and real-time PCR (polymerase chain reaction), have been investigated as potential tools for the timely diagnosis and management of neonatal sepsis, although these methods are expensive and not available in all medical settings.

Antibiotic resistance is an additional problem to address when choosing the right treatment, as it can further complicate the management of neonatal sepsis.

Culture-negative neonatal sepsis remains a significant challenge for the neonatologist, since the time elapsed between the moment sepsis is suspected and the initiation of empirical therapy can make the difference between survival and death. Continued efforts are needed to develop more reliable and effective diagnostic tools for timely and appropriate intervention and to identify alternative treatment strategies beyond traditional antimicrobial therapies.

Our study aimed to assess the influence of risk factors and the utility of currently used biomarkers in culture-negative neonatal EOS. We looked for a new evidence-based approach to infants at risk for developing EOS that would lead to a more standardized and limited use of antibiotics.

Our objective was to identify a better tool to distinguish between suspected EOS and culture-negative confirmed EOS, thereby reducing the incidence of unnecessary antibiotic exposure.

## 2. Materials and Methods

### 2.1. Study Design

A retrospective study, including preterm and term newborns at risk for EOS admitted to the Neonatal Intensive Care Unit (NICU) in our neonatal tertiary unit at the University Emergency Hospital of Bucharest for 12 months, from January 2021 to January 2022, was conducted.

The neonates included in our research were classified into two groups: confirmed negative-culture early-onset sepsis (CN-EOS) and suspected early-onset sepsis (S-EOS). Patients from both groups received antibiotic therapy from the first day of life; the type and duration of the antibiotic therapies were different in the two groups.

We collected the demographic characteristics and the laboratory records from the medical database of patients, and the medical charts were retrospectively reviewed for screening and diagnostic parameters of neonatal sepsis.

Before inclusion in the study, informed consent was obtained from the mothers of all the newborns after assuring that they completely understood the protocol and participated voluntarily in the research.

### 2.2. Inclusion Criteria and Definitions

A total of 131 neonates were enrolled in this retrospective single-center study. The cases included were patients with CN-EOS and S- EOS born in our hospital within a 12-month period who received antibiotic therapy after a complete screening for suspicion of neonatal sepsis, according to our unit protocol.

Neonatal sepsis was defined based on NICE guidelines (www.nice.org.uk/guidance/ng195, accessed on 5 July 2021) and the criteria of the Hospital’s Epidemiology Surveillance Department. The following signs and symptoms were considered inclusion criteria: temperature instability (<36.5 or >38 °C degrees), recurrent apneas, increased respiratory or oxygen support, cardio–circulatory impairment (hypotension, bradycardia, tachycardia), acidosis, changes in peripheral perfusion, hypoglycemia, hyperglycemia, feeding intolerance with gastrointestinal signs, neurological impairment (hypotonia, irritability, lethargy), abnormal laboratory parameters (CRP > 10 mg/L, PCT > 10 ng/mL, WBC). The clinical and laboratory findings for neonatal sepsis are included in Table 1.

Positivity in at least two of the clinical categories and at least two of the laboratory categories is considered clinical sepsis; it can be used up to 44 weeks postnatal.

Confirmed early neonatal sepsis with negative cultures (clinical sepsis) included newborns with at least two symptoms or signs of sepsis, according to internal and international guidelines, and/or abnormal values of serum biomarkers and required at least five days of antimicrobial therapy.

Suspected early neonatal sepsis with negative cultures included newborns with perinatal demonstrated risk factors, mild clinical expression, with/without alterations of biochemical markers, and received less than five days of antibiotic therapy.

For all the patients, CRP, PCT and WBC were measured, and a blood culture was tested at birth. The cutoff value for CRP levels was above 10 mg/L and for PCT > 10 ng/mL.

A second sample for CRP and PCT measurement was repeated after 72 h. Antibiotic therapy was stopped if there were no clinical signs and the second measurements of CRP and PCT were normal. These neonates were included in the S-EOS group. The rest of the neonates that continued antibiotic therapy due to clinical signs and/or abnormal values of serum biomarkers at 72 h of life, even with a negative blood culture, were considered confirmed CN-EOS.

From a total of 131 cases of included neonates, 66 cases were assigned to the confirmed EOS group (CN-EOS cases) and 65 cases to the suspected EOS group (S-EOS).

### 2.3. Statistical Analysis

Sample size calculation was assessed with G*Power 3.1.9.7 software (https://www.psychologie.hhu.de/arbeitsgruppen/allgemeine-psychologie-und-arbeitspsychologie/gpower, accessed on 15 January 2024), based on a convenience sampling method. For a medium effect size (*f* = 0.25), an alpha level of α = 0.05 and a power of 1-β = 0.80, a minimum sample size of 64 participants was needed.

The statistical analysis was conducted in SPSS software, version 25.0. Categorical variables were presented as frequencies and percentages, while for the continuous variables, descriptive statistics indicators for data summarization, namely, the mean (±standard deviation) were used. The normality of the continuous variables was evaluated with the Shapiro–Wilk test. The differences between the continuous variable groups were determined by Student’s *t*-test or the Mann–Whitney U test, depending on the normality of their distributions, whereas for the categorical variables, the Chi-square test (X^2^) was employed. Logistic regression was used to identify the risk factors for confirmed EOS, and the performance of laboratory biomarkers (CRP and PCT) in diagnosing confirmed EOS was calculated by using the area under the curve (AUC) of the receiver operating curve (ROC). More exactly, the confirmed EOS was assessed by CRP and PCT at different timeframes by reporting the AUC, sensitivity, specificity and best cut-off values. All the tests were statistically significant at a *p*-value < 0.05.

## 3. Results

### Newborn Baseline Characteristics

This study included 131 newborns admitted in the NICU with suspicion of early neonatal sepsis due to the presence of maternal risk factors or clinical signs and symptoms, as depicted in Table 2.

We extracted data from the patients’ medical files regarding the method of delivery and Apgar score as a marker of success in transition from the intrauterine to extrauterine environment. The results are illustrated in Table 3.

The differences between the EOS groups considering the maternal characteristics (demographic info, medical status during pregnancy, socio-economic status) are illustrated in Table 4.

A comparison between the two groups when it comes to clinical signs of EOS can be found in Table 5.

All the patients’ biochemical data were analyzed to identify the differences between the CN-EOS and S-EOS groups. The WBC, hemoglobin level, hematocrit level, neutrophil percent, CRP, PCT and peripheral cultures are compared in Table 6.

There were more NICU days, more overall hospitalization days and an increased need for NICU-specific treatment observed in the CN-EOS group, as can be observed in Table 7.

The maternal age, the gestational age of the neonates, the birth weight, the Apgar score and the premature rupture of membranes (PROM) of more than 18 h were proved to be risk factors for confirmed EOS (Table 8).

During hospital stays, more elements were connected to a higher incidence of CN-EOS. These factors connected with CN-EOS are depicted in Table 9.

**Figure 1:**
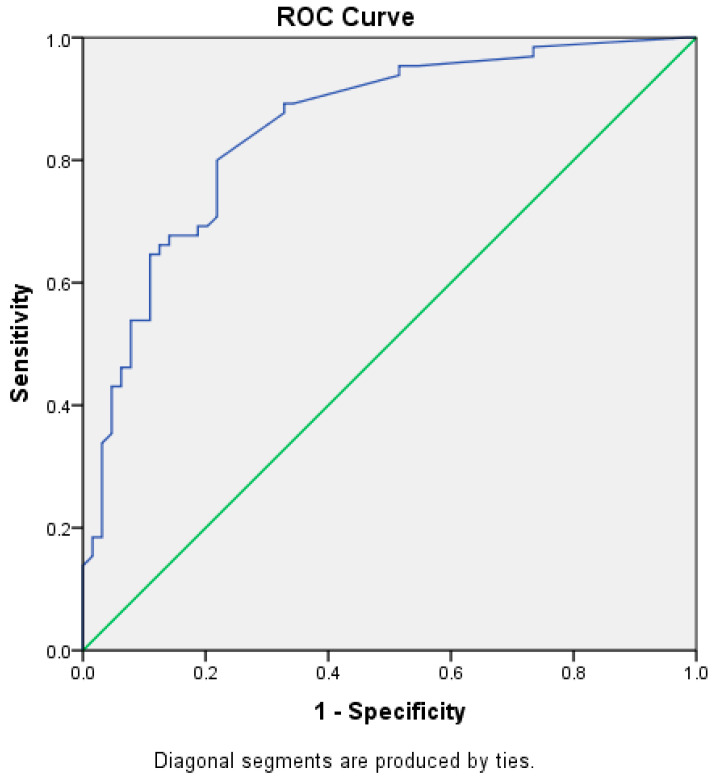
The ROC curve of PCT at 72 h for the confirmed EOS.

The CRP measurements of 24 h and 72 h were not identified as statistically significant predictors of confirmed EOS. As for the measurements of PCT, the findings differed. The PCT measurement at 24 h was not statistically significant, but the PCT at 72 h was a satisfactory predictor of confirmed EOS (AUC = 0.85, *p* = 0.001, 95% CI 0.78–0.91). The best cut-off value was 0.27, at a sensitivity of 93.8% and a specificity of 51.6% (Figure 1).

## 4. Discussions

Neonatal sepsis poses a significant challenge in clinical practice, particularly when the causative microorganism cannot be identified through traditional blood culture methods [21]. Despite constant progress in modern medicine and continuous focus on early detection and treatment of neonatal sepsis, the mortality rate remains extremely high, up to 25% [22,23]. According to different researchers, 84% of deaths caused by neonatal sepsis can be prevented by improving the understanding of the risk profile, followed by prompt intervention [24]. Despite numerous investigations, the etiology of EOS remains unknown in many cases, and the systemic infection’s pathogenesis is still poorly understood [25]. Negative-culture sepsis presents an additional challenge, as management of each case should be adapted based on individual interchanging data and personal experience. Severe culture-negative sepsis accounts for a significant proportion of neonatal sepsis cases, with estimates ranging from 28% to 49% [26,27]

Regarding maternal risk factors and incidence of EOS, an international survey conducted by van Herk et al. demonstrated that, worldwide, there is a lack of consensus in the guidelines regarding the management of well-appearing infants with risk factors only, leading to great variability in the management of these newborns, unlike those with risk factors and clinical signs, where there is a general acceptance of not delaying empiric antibiotic therapy [28].

The primary aim of this research was to determine potential predictors for the CN-EOS among maternal risk factors, clinical signs and elevated values of currently used biomarkers, CRP, PCT and WBC, to facilitate early and accurate diagnosis, thus guiding appropriate antibiotic therapy.

Our study confirms the importance of maternal risk factors (PROM > 18 h, mother’s age, smoking during pregnancy) for predicting EOS. Cases of births with PROM > 18 h had a 9.67-fold increase in neonatal sepsis. Several previous studies investigated the impact of PROM, and there is evidence that it was significant, especially in cases of uninvestigated pregnancies [29]. In our study, among the CN-EOS group, 81.8% of infants were born to mothers with a urogenital infection or uninvestigated pregnancies, of which chorioamnionitis diagnosis was established in only 28.8%, a significantly higher incidence compared to 9.2% in the S-EOS group. The current literature suggests that only 1–10% of pregnancies are complicated by clinical chorioamnionitis, a diagnosis made by clinical criteria, which hardly differentiates inflammation from infection [30]. Subsequently, many well-appearing infants are exposed to empirical antibiotherapy for the prevention or treatment of suspected sepsis [31]. Experts’ opinions from NICHD (National Institute of Child Health and Human Development) recommended using “intrauterine inflammation, infection (triple I)” instead of “chorioamnionitis” [30]. A recent meta-analysis conducted by Beck et al. evidenced that histological chorioamnionitis was associated with a higher incidence of EOS compared to clinical chorioamnionitis, at 7% versus 6% [32].

It has been demonstrated that antibiotics used during pregnancy cross the placenta into the fetus’ circulation, raising concerns about the accuracy of blood cultures and asymptomatic bacteremia, leading to an increase in the number of mothers and newborns receiving antibiotics [33]. In our study, more than 35% of mothers were exposed to perinatal antibiotherapy. A recent study conducted in Germany highlighted the importance of decreasing prenatal exposure to antibiotics due to implementing new stewardship programs and reported a recent decrease in the rates of antibiotic resistance of Escherichia coli [34]. Kuzniewicz and Puopolo conducted extensive research in Northern California that included 204,485 infants, aiming at the utility of elaborating a quantitative model to assess the indication for initiating empiric antibiotherapy. The results demonstrate that by using such a model, which included the degree of prematurity, maternal fever and duration of PROM, there might be a significant decrease in the number of newborns treated with antibiotics [35].

In our study, low birth weight and gestational age can be considered potential risk factors for EOS because there were significant differences between the two groups. In the CN-EOS group, the average birth weight was 1998.56 g compared to 3125.43 g in the S-EOS group, suggesting that intrauterine infection interferes with weight gain and/or low-birth-weight infants are more susceptible to pathogens. Regarding low-birth-weight infants, a meta-analysis of 240 studies conducted by Gan M.Y. et al. in 2022 evidenced a significant difference regarding the risk of mortality due to sepsis between patients with a birth weight < 1500 g and newborns with a birth weight > 2500 g, at a rate of 24% versus 15% [24]. Accordingly, another study conducted by Liang L et al. in Canada showed a six-fold increase in terms of sepsis and associated mortality in low-birth-weight newborns [36].

In our study, the mean gestational age was lower in the CN-EOS group, 32.50 weeks (SD ± 4.52) compared to 38.52 weeks (SD ± 1.60) in the suspected EOS group. The immune system of preterm infants is distinct and contributes to their higher risk for developing infections and sepsis [37]. Premature birth is often determined by an intrauterine infection; overproduction of prostaglandins caused by inflammation leads to uterine contractions, contributing to preterm delivery [38].

A study that included 172 neonates with sepsis showed that premature infants (gestational age < 29 weeks of gestation) had a 65.7% mortality rate associated with EOS compared to higher gestational age groups [14]. Nevertheless, in preterm infants, it is rather difficult to focus on the independent contribution of any specific risk factors for EOS, besides gestational age [39].

In terms of gender, we identified no difference between the male and female ratio among our patients, although there are studies that evidenced that the male gender is more likely to be at risk for EOS [40], despite others that reveal female predominance or an equal ratio between genders [41].

An important finding in our research was revealed after analyzing the Apgar scores at birth. The mean Apgar score at 1 and 5 min was lower in the CN-EOS group compared to the suspected EOS group, with a mean Apgar score of 6.74 (SD ± 1.87) at 1 min and 7.80 (SD ± 1.39) at 5 min compared to the S-EOS group, where the mean values were 8.91 (SD ± 0.86) at 1 min and 9.45 (SD ± 0.61) at 5 min, with a statistically significant difference, as shown in Table 3. This correlation can lead to the conclusion that antenatal infection can make the transition to an extrauterine environment more difficult.

In our research, the incidence of newborns with respiratory distress syndrome and apnea (*p* = 0.001, OR = 62.17) and feeding intolerance (*p* = 0.008, OR = 3.68) was significantly higher in the CN-EOS group compared to suspected EOS cases, as shown in Table 7 and Table 8. Furthermore, we found that pneumothorax, seizures and bleeding episodes displayed a pronounced difference (*p* = 0.001) in the CN-EOS group versus the S-EOS group.

After a thorough analysis of the laboratory parameters, we found some noteworthy observations. Across the CBC components, WBC, neutrophils and platelet count exhibited significant differences. Newborns with CN-EOS had lower WBC counts (14.165 × 10^9^/L) compared to the infants with suspected EOS (16.986 × 10^9^/L), with a *p*-value of 0.03. Neutrophil and platelet count presented statistically significant differences (*p* = 0.02 and *p* = 0.001, respectively) between the two groups, as shown in Table 6. Serum lactate was slightly higher in the CN-EOS group but did not show a difference after statistical analysis.

Another significant finding in the CN-EOS group was a lower level of hemoglobin and hematocrit during the first 24 h of life compared to the suspected group, with statistically significant differences (*p* = 0.03, OR = 0.85 for hemoglobin and *p* = 0.04, OR = 0.95 for hematocrit). We found that hypoglycemia showed an association with CN-EOS, with a *p*-value of 0.02 and OR = 2.88, CI = 1.15–7.16.

The role of inflammatory biomarkers in the diagnosis and management of culture-negative neonatal sepsis has been the subject of growing interest. PCT is a more specific marker of bacterial infection compared to traditional inflammatory markers [42]. CRP is a general marker of inflammation and may not be as specific in differentiating between sepsis and other inflammatory conditions, particularly in the postoperative setting [42]. 

Research has demonstrated that PCT levels rise more rapidly and are more sensitive in detecting bacterial infections compared to other biomarkers, such as CRP [43]. Studies have found that procalcitonin has the greatest sensitivity and specificity for distinguishing between patients with systemic inflammatory response syndrome and those with sepsis, including in the context of postoperative inflammation [42]. 

In our research, we identified 76.9% of neonates with CRP levels above 10 mg/L at 24 h of life in the S-EOS group, three times more than the number of neonates in the EOS group with CRP > 10 mg/L (23.1%). This finding is consistent with other data reported in the current literature that emphasize the physiological elevation of CRP in neonates during the first days of life [44]. Likewise, Mjelle et al. concluded in their research on healthy-term newborns that 1 in 14 had CRP levels > 20 mg/L and physiological elevation was mostly influenced by several factors, such as active labor [44,45]. The link between elevated CRP levels and stress during birth was also observed by Vogl et al. in research conducted in Austria [46]. Our study shows 46.2% of vaginal delivery in the neonates with elevated CRP compared to only 29.5% in the neonates with a normal CRP value. The similarity with existing evidence can explain the small number of neonates with elevated CRP levels in the EOS group. Recent findings presented by Xiaojuan Li et al. show moderate predictive accuracy for neonatal sepsis, AUC = 0.68 [47].

We underline the importance of early recognition of the difference between the physiological and inflammatory increase of CRP in the first days of life to significantly decrease antibiotic exposure and prevent drug resistance.

Numerous studies have explored the utility of PCT for accurately identifying and differentiating bacterial infections from other inflammatory conditions, as well as its potential to guide the initiation and duration of antimicrobial therapy [48,49]. It is known that there is a physiological increase in PCT values during the first 48 h of life determined by various factors (hypoxia, pre-eclampsia, maternal infections, perinatal asphyxia) [50].

Current research evidences that PCT is more accurate and detects EOS significantly sooner compared to CRP [51]. A normal result of PCT has a significant negative predictive value for neonatal sepsis in infants with a gestational age > 34 weeks [13].

Our study revealed a higher number of patients in the CN-EOS group with PCT > 10 ng/L at 24 h of life compared to the suspected group, without significant differences (*p*-value = 0.07). Furthermore, an assessment of the PCT values at 72 h of life demonstrated that among the CN-EOS group, 75.75% of infants had PCT > 0.5 ng/L, unlike the suspected EOS group, which had markedly fewer patients with PCT > 0.5 ng/L (*p*-value = 0.001, AUC = 0.85, 95%CI = 0.78–0.91), indicating a persistent inflammatory response.

Consistent with the data reported in the literature, our results show that standard laboratory tests (CBC, CRP, PCT) lack specificity due to poor positive predictive value and should not be used alone to diagnose early neonatal sepsis.

Recent findings regarding the best approach in stratifying EOS risk evidenced that none of the actual methods for identifying newborns with a sepsis risk have an acceptable sensitivity [52]; therefore, improvements in this field must be continued for a higher accuracy in EOS prediction [53,54]. Since a positive blood culture remains the gold standard for accurate diagnosis of sepsis, a negative blood culture remains a challenge for all neonatologists who strive to distinguish truly infected infants from sepsis-like conditions. There is ongoing interest regarding the reasons for the high number of negative results, and the blood volume is an important factor that contributes to an accurate result [55]. Actual recommendations for neonates are that a minimum of 0.5 mL, ideally 1 mL, of blood is required for optimal results (sensitivity of blood culture decreases up to 40% if only 0.5 mL is collected) [56]. Coggins et al. demonstrated in their research that a better strategy that could improve the rate of positive blood cultures would be collecting a second blood sample from a different site, at the same time, although in the case of extremely low birth weight infants, it could be a difficult procedure [57]. A recent study that included 3665 newborns stated that by including anaerobic cultures in the NICU routine identification process, pathogens that are not evidenced by aerobic samplings could be detected [58]. In our study, the patients with CN-EOS and negative blood cultures were tested at birth; we did not collect a second sample or an anaerobic one. A study developed in the UK by Blackburn et al. demonstrated that among all blood cultures collected in neonates during the research, 50% were obtained in the first 24 h of life and only 0.8% showed positive results [59]. It is important to note that we should also focus on neonates with mothers who received intrapartum prophylaxis with antibiotics. Are they at higher risk or, on the contrary, are they protected by the bactericidal effect of antibiotics that cross the placenta, pretreating those infants? Paradoxically, the exact opposite happens, and these newborns are treated more intensively [60].

## 5. Limitations

Our study, although containing comprehensive insights regarding culture-negative neonatal sepsis, has several limitations. It relies on the experience of single-center research with a relatively small number of patients, and therefore, it prevents us from generalizing the conclusions to other centers whose demographics and clinical practices differ from ours. Another important limitation is that it is a retrospective study, and patient information was collected before establishing the intention for this research; therefore, some incomplete correlations might occur. Given the fact that our country has a mid-level medical care system, and the risk of neonatal EOS might be higher than in high-income countries, we limit the applicability of our results to similar medical centers. Unfortunately, we were unable to document the GBS status for all the mothers, leading to a lack of important correlation to CN-EOS risk factors.

## 6. Conclusions

A thorough understanding of culture-negative neonatal sepsis and risk stratification will help neonatologists improve the quality of care provided in the NICU. The first step could be the elaboration of a more standard definition of neonatal sepsis that could limit the subjective variations of the management and therefore guide the appropriate antibiotic use. Second, neonatologists should not rely on biomarkers alone but should use them in combination with repeated clinical evaluations and newer methods of blood culture identification to decrease irrational administration of antibiotics out of fear of this life-threatening condition, neonatal sepsis.

Exposure to antibiotherapy from the first days of life and its subsequent consequences has raised great concerns worldwide. Great efforts have been made to ensure more accurate identification of the real infected cases to prevent unnecessary treatment of the healthy ones.

## Figures and Tables

**Table 1:** Clinical and paraclinical criteria for neonatal sepsis.

Clinical Findings	Laboratory Findings
Body temperature (temperature irregularities, >38 °C, <36 °C)	Leukocyte count (>20.000/mm^3^ or <4000/mm^3^)
Cardiovascular instability (tachycardia or bradycardia and/or rhythm irregularity; urine amount < 1 mL/kg/h; hypotension; impaired peripheral perfusion)	Immature/total neutrophil ratio (≥0.2)
Skin and subcutaneous lesions (petechiae, scleredema)	Platelet count (<100.000/mm^3^)
Respiratory instability (apnea or tachypnea or increased oxygen demand or increased need for ventilation support)	CRP > 15 mg/L (1.5 mg/dL) or PCT ≥ 2 ng/mL
Gastrointestinal issues (nutritional intolerance, insufficient breastfeeding, abdominal distension)	In blood sugar monitoring at least twice (hyperglycemia > 180 mg/dL or 10 mMoL/L or hypoglycemia < 45 mg/dL or 2.5 mMoL/L)
Non-specific symptoms (irritability, lethargy, hypotonia)	Metabolic acidosis (base deficit < 10 mEq/L or serum lactate > 2 mMoL/L)

(Adapted from [20]).

**Table 2:** Baseline characteristics of the newborns included in this study.

Variable		CN-EOS (*n* = 66) Mean ± SD/Freq. (%)	S-EOS (*n* = 65) Mean ± SD/Freq. (%)	*p*-Value
Gestational age		32.50 (±4.52)	38.52 (±1.60)	0.001
Birth weight		1998.56 (±920.02)	3145.23 (±553.13)	0.001
Length		42.68 (±5.82)	49.04 (±2.24)	0.001
Head circumference		29.54 (±3.99)	33.13 (±1.56)	0.001
Ponderal index		2.37 (±0.37)	2.63 (±0.26)	0.001
Gender	Male	37 (56.1%)	37 (56.9%)	0.92
	Female	29 (43.9%)	28 (43.1%)	

Note: SD—standard deviation.

**Table 3:** Method of delivery and Apgar score of the patients included in this study.

Variable		CN-EOS (*n* = 66) Mean ± SD/Freq. (%)	S-EOS (*n* = 65) Mean ± SD/Freq. (%)	*p*-Value
APGAR 1 min		6.74 (±1.87)	8.91 (±0.86)	0.001
APGAR 5 min		7.80 (±1.39)	9.45 (±0.61)	0.001
Vaginal delivery	Yes	21 (31.8%)	22 (33.8%)	0.80

**Table 4:** Comparison of maternal characteristics and clinical findings between the confirmed EOS group and the suspected EOS group.

Maternal Characteristics and Clinical Findings		CN-EOS (*n* = 66) Mean ± SD/Freq (%)	S-EOS (*n* = 65) Mean ± SD/Freq (%)	*p*-Value
Age		30.35 (±6.42)	28.17 (±5.87)	0.04
Investigated pregnancy		43 (65.2%)	43 (66.2%)	0.90
Antibiotherapy during pregnancy		25 (37.9%)	22 (33.8%)	0.63
Corthicotherapy for the risk of premature birth		17 (±25.8%)	6 (±9.2%)	0.01
PROM > 18 h		38 (57.6%)	8 (12.3%)	0.001
Urogenital infections during pregnancy		29 (43.9%)	31 (47.7%)	0.54
Alcohol consumption during pregnancy		8 (12.1%)	14 (21.5%)	0.14
Smoking during pregnancy		40 (61.5%)	20 (30.3%)	0.001
Socio-economic level	Low	17 (25.8%)	24 (36.9%)	0.36
	Medium	34 (51.5%)	27 (41.5%)	
	High	15 (22.7%)	14 (21.5%)	
Leukocyte count		12,854.5 (±4244.31)	11,554.7 (±3282.1)	0.052
Neutrophil %		70.15 (±11.83)	73.20 (±11.34)	0.13
CRP		19.71 (±43.12)	7.69 (±9.64)	0.32
Fibrinogen		580.86 (±136.4)	516.02 (±112.85)	0.004

**Table 5:** Comparison of newborns’ clinical signs between the confirmed EOS group and the suspected EOS group.

Newborns’ Clinical Signs	CN-EOS (*n* = 66)/Freq. (%)	S-EOS (*n* = 65)/Freq. (%)	*p*-Value
Respiratory distress syndrome	53 (80.3%)	4 (6.2%)	0.001
Pneumothorax	8 (12.1%)	0 (0%)	0.004
Apnea	44 (66.7%)	3 (4.6%)	0.001
Seizures	10 (15.2%)	0 (0%)	0.001
Bleeding	21 (31.8%)	2 (3.1%)	0.001
Feeding intolerance	18 (27.3%)	6 (9.2%)	0.008

**Table 6:** Comparison of laboratory blood tests between newborns with confirmed EOS and the suspected EOS group.

Newborns’ Laboratory Values	CN-EOS (*n* = 66) Mean ± SD/Freq. (%)	S-EOS (*n* = 65) Mean ± SD/Freq. (%)	*p*-Value
Leukocyte count—first 24 h	14,165 (±9248.17)	16,986.15 (±5225.71)	0.03
Hemoglobin level—first 24 h	15.24 (±2.67)	16.14 (±2.13)	0.03
Hematocrit level—first 24 h	45.92 (±7.94)	48.56 (±6.53)	0.04
Platelet count—first 24 h	238,984.85 (±75,998.07)	289,292.31 (±66,471.02)	0.001
Neutrophil %—first 24 h	51.34 (±18.34)	59.89 (±12.43)	0.002
Serum lactate—first 24 h	3.36 (±2.09)	2.59 (±1.15)	0.34
Hypoglycemia	19 (28.8%)	8 (12.3%)	0.02
Positive peripheric cultures	16 (24.63%)	0 (0%)	0.001
CRP at 24 h	7.28 (16.2)	10.94 (±14.33)	0.36
PCT at 24 h	23.19 (±21.54)	16.91 (±17.38)	0.07
CRP at 72 h	5.19 (±10.64)	3.49 (±5.35)	0.70
PCT at 72 h	5.57 (±5.86)	1.01 (±2.07)	0.001

**Table 7:** Comparison of newborns’ therapeutic interventions between the confirmed EOS group and the suspected EOS group.

Newborns’ Therapeutic Strategies	CN- EOS (*n* = 66) Mean ± Standard Deviation	S- EOS (*n* =65) Mean ± Standard Deviation	*p*-Value
NICU admission (days)	20.26 (27.04)	0.45 (1.46)	0.001
Hospitalization time (days)	33.76 (33.00)	5.23 (3.66)	0.001
Duration of invasive mechanical ventilation (h)	83.00 (211.86)	0.00 (0.00)	0.001
Duration of oxygen therapy (h)	214.36 (464.68)	1.21 (4.45)	0.001
Duration of endovenous perfusion (days)	14.78 (17.84)	0.26 (0.85)	0.001
Duration of total parenteral nutrition (days)	5.59 (9.42)	0.07 (0.36)	0.001
Total duration of antibiotic therapy (days)	18.06 (17.08)	3.32 (1.14)	0.001
Need for inotropic therapy	3.44 (7.37)	0.00 (0.00)	0.001
Fresh frozen plasma transfusion	0.80 (1.53)	0.00 (0.00)	0.001
Erythrocyte transfusion	1.27 (2.41)	0.00 (0.00)	0.001
Phototherapy	28.39 (27.25)	5.08 (12.33)	0.001
IVIG administration	6 (9.1%)	0 (0%)	0.01
Sedation	24 (36.4%)	0 (0%)	0.001

**Table 8:** The potential risk factors of CN-EOS.

Risk Factor	OR, 95% CI, *p*
Mother’s age	OR = 1.06, 1.00–1.12, *p* = 0.04
Gestational age	OR = 0.52, 0.41–0.66, *p* = 0.001
Birth weight	OR = 0.99, 0.99–0.98, *p* = 0.001
PROM > 18 h	OR = 9.67, 3.98–23.46, *p* = 0.001
Body temperature on NICU admission	OR = 6.17, 1.56–24.39, *p* = 0.009
APGAR at 1 min	OR = 0.20, 0.11–0.36, *p* = 0.001
APGAR at 5 min	OR = 0.09, 0.04–0.22, *p* = 0.001

**Table 9:** Elements connected to a higher incidence of CN- EOS during the hospital stay.

Risk Factor	OR, 95% CI, *p*
Low levels of hemoglobin at 24 h	OR = 0.85, 0.73–0.99, *p* = 0.04
Low hematocrit at 24 h	OR = 0.95, 0.90–0.99, *p* = 0.04
Low level of neutrophil count	OR = 0.96, 0.94–0.98, *p* = 0.004
Hypoglycemia	OR = 2.88, 1.15–7.16, *p* = 0.02
Respiratory distress syndrome	OR = 62.17, 19.11–202.24, *p* = 0.001
Apnea	OR = 41.33, 11.64–146.68, *p* = 0.001
Bleeding	OR = 14.70, 3.28–65.88, *p* = 0.001
Total parenteral nutrition (days)	OR = 7.88, 3.27–18.96, *p* = 0.001
Meropenem as first-line antibiotherapy	OR = 3.49, 1.25–9.73, *p* = 0.01
Antibiotherapy duration	OR = 4.36, 2.41–7.89, *p* = 0.001
NICU stay (days)	OR = 2.01, 1.56–2.58, *p* = 0.001

Note: OR—odds ratio, CI—confidence interval.

## Data Availability

The original contributions presented in this study are included in the article. Further inquiries can be directed to the corresponding authors.
